# The Regulation of Monoamine Oxidase A Gene Expression by Distinct Variable Number Tandem Repeats

**DOI:** 10.1007/s12031-018-1044-z

**Published:** 2018-03-14

**Authors:** Maurizio Manca, Veridiana Pessoa, Ana Illera Lopez, Patrick T. Harrison, Fabio Miyajima, Helen Sharp, Andrew Pickles, Jonathan Hill, Chris Murgatroyd, Vivien J. Bubb, John P. Quinn

**Affiliations:** 1 Department of Molecular and Clinical Pharmacology, Institute of Translational Medicine, University of Liverpool, Liverpool, L69 3BX UK; 2 Institute of Psychology, Health and Society, University of Liverpool, Liverpool, UK; 3 Present Address: Biomarker Research Laboratory, University of Huddersfield, Queensgate, Huddersfield, HD1 3DH UK; 4 Present Address: Department of Physiology and Pharmacology, Faculty of Medicine, Drug Development and Research Center, Federal University of Ceara, Fortaleza, Ceara Brazil; 5 Department of Physiology, BioSciences Institute, University College Cork, Cork, Ireland; 6 King’s College London, MRC Social Genetic and Developmental Psychiatry Research Centre, Institute of Psychiatry, London, UK; 7 School for Psychology and Clinical Language Sciences, University of Reading, Reading, UK; 8 School of Healthcare Science, Manchester Metropolitan University, Manchester, UK

**Keywords:** *MAOA*, Isoforms, VNTR, Gene expression, Transcription, Haplotype

## Abstract

The monoamine oxidase A (*MAOA*) uVNTR (upstream variable number tandem repeat) is one of the most often cited examples of a gene by environment interaction (GxE) in relation to behavioral traits. However, *MAOA* possesses a second VNTR, 500 bp upstream of the uVNTR, which is termed d- or distal VNTR. Furthermore, genomic analysis indicates that there are a minimum of two transcriptional start sites (TSSs) for *MAOA*, one of which encompasses the uVNTR within the 5′ untranslated region of one of the isoforms. Through expression analysis in semi-haploid HAP1 cell lines genetically engineered in order to knockout (KO) either the uVNTR, dVNTR, or both VNTRs, we assessed the effect of the two *MAOA* VNTRs, either alone or in combination, on gene expression directed from the different TSSs. Complementing our functional analysis, we determined the haplotype variation of these VNTRs in the general population. The expression of the two *MAOA* isoforms was differentially modulated by the two VNTRs located in the promoter region. The most extensively studied uVNTR, previously considered a positive regulator of the *MAOA* gene, did not modulate the expression of what it is considered the canonical isoform, while we found that the dVNTR positively regulated this isoform in our model. In contrast, both the uVNTR and the dVNTR were found to act as negative regulators of the second less abundant *MAOA* isoform. The haplotype analysis for these two VNTRs demonstrated a bias against the presence of one of the potential variants. The uVNTR and dVNTR differentially affect expression of distinct *MAOA* isoforms, and thus, their combined profiling offers new insights into gene-regulation, GxE interaction, and ultimately *MAOA*-driven behavior.

**Electronic supplementary material:**

The online version of this article (10.1007/s12031-018-1044-z) contains supplementary material, which is available to authorized users.

## Introduction

**Fig. 1:**
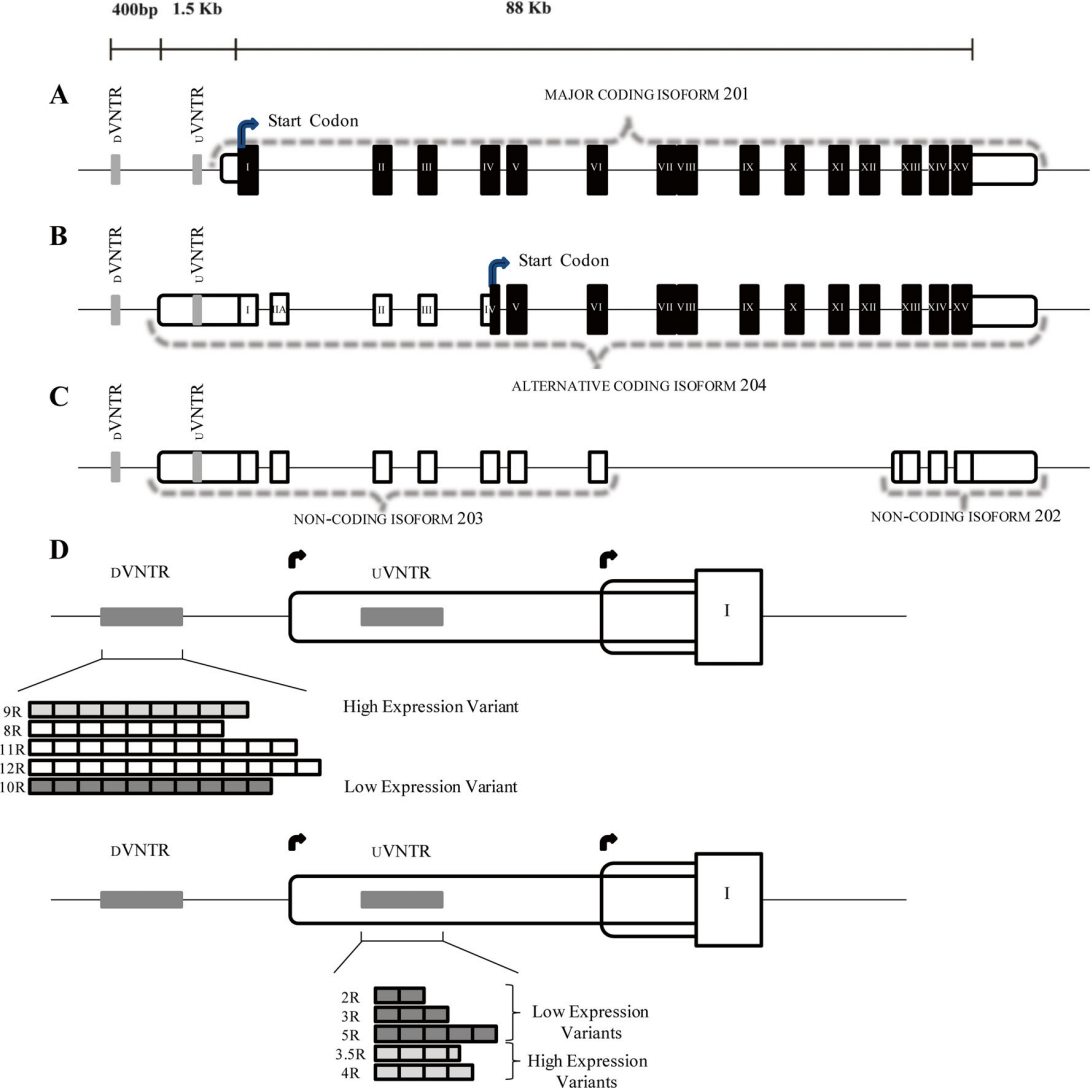
Monoamine oxidase A (*MAOA*) isoforms. Graphic representation of the *MAOA* gene as reported in UCSC genome browser Hg38 and the most recent version of the Hg19. White rounded boxes represent 5′ and 3′ UTRs, and black boxes the translated exons. Curved black arrows indicate the translational start sites. Gray bars are the dVNTR and the uVNTR from left to right respectively. **a** Major *MAOA* isoform; NCBI accession number: BC008064 (Version BC008064.2), Ensembl version 201. **b** MAOA secondary isoform; NCBI accession number: AK293926 (Version AK293926.1), Ensembl version 204. **c** Untranslated processed transcripts Ensembl version 203 and 202 respectively. **d** *MAOA* dVNTR genotyped alleles: 8R to 12R. The structure forming the dVNTR is represented above the squares, each of which represents a repeat unit. 9R represents high expression, 10R low expression, 8-11-12R the other copy variants as reported by (Philibert et al. 2011). MAOA uVNTR genotyped alleles: 2R to 5R. 2R, 3R, and 5R are the low expression variants, and 3.5R and 4R the high expression variants as first reported by Sabol et al. (1998)

Monoamine oxidase A (*MAOA*), a major regulator of monoamine neurotransmitters in the brain, is one of the best characterized and most cited genes in gene × environment interaction (GxE) studies, particularly in relation to central nervous system (CNS) disorders (Nikulina et al. 2012; Philibert et al. 2008; Reif et al. 2014; Samochowiec et al. 2004) and behavioral traits (Aslund et al. 2011; Caspi et al. 2002; Chester et al. 2015; Hill et al. 2013; Pickles et al. 2013). Two coding transcripts for the *MAOA* gene have been reported, with transcriptional start sites (TSSs) separated by approximately 1.3 kb, resulting in two putative coding isoforms with distinct 5′ untranslated regions (5′ UTRs). These isoforms vary in length and contain alternative exons and distinct start codons which potentially lead to the translation of two protein isoforms (Fig. 1a, b); however, the functional consequences of this have not been discussed in the literature. The regulation of these two TSSs is expected in part to be directed by two distinct variable number tandem repeat (VNTR) domains identified in the *MAOA* promoter region, which have previously been demonstrated to support gene expression in reporter gene assays (Philibert et al. 2011; Sabol et al. 1998). The first, termed uVNTR, is located 1 kb upstream of the TSS for what we will term the primary *MAOA* isoform (Fig. 1a; Ensembl isoform 201), which is the most abundant mRNA and the TSS most referred to in previous communications analyzing the function of the uVNTR (Edwards et al. 2010; Lee and Ham 2008; Lung et al. 2011; Reif et al. 2008; Sabol et al. 1998). In this context, the uVNTR is considered a transcriptional regulatory domain upstream of this major TSS. However, in the second isoform, it is transcribed in the 5′UTR (Fig. 1b), which is produced from the more 5′ TSS. The *MAOA* uVNTR consists of a 30-bp motif that can be repeated 2, 3, 3.5, 4, and 5 times (Sabol et al. 1998). The 2, 3, and 5 repeats are generally defined as low expression variants (MAOA-L), while the 3.5 and 4 repeat VNTRs have been shown to demonstrate a 2- to 10-fold increase in reporter gene expression and considered high expression variants (MAOA-H) (Sabol et al. 1998). We and others have previously reported evidence that specific uVNTR variants act as a moderators of the association observed between certain environmental risk factors and child behavioral problems (Fergusson et al. 2012; Hill et al. 2013; Pickles et al. 2013). However, the majority of the literature refers only to the uVNTR as the major, or sole, mediator of *MAOA* expression and a possible biomarker for stress-related illnesses, with the different alleles implicated in major depressive disorder, addiction, violent behavior (Fan and Sklar 2005; Frazzetto et al. 2007; Melas et al. 2013; Philibert et al. 2008; Reif et al. 2014), suicide attempts in bipolar females (Ho et al. 2000), and depressed males (Du et al. 2002). The second VNTR, termed dVNTR, is located approximately 500 bp upstream of the uVNTR and is composed of two different decamer repeats CCCCTCCCCG (repeat A) and CTCCTCCCCG (repeat B) (Philibert et al. 2011). Genotypes of 8, 9, 10, 11, and 12 repeats have been documented, with 9R and 10R being the most common. In reporter gene assays, similarly to the uVNTR, the 9R and 10R differ in transcriptional efficiency, where the 9R is stronger than the 10R and the other genotypes are intermediate (Philibert et al. 2011).

Through analysis of the semi-haploid HAP1 cell line (Carette et al. 2011) deleted for either the uVNTR, dVNTR, or both VNTRs, we assessed the effect the two VNTRs, combined and individually, on *MAOA* expression and specifically on the two distinct TSSs. We also analyzed the variation in the haplotype of the *MAOA* promoter in the general population to determine common haplotypes that may be used for further stratification of the genetic risk of *MAOA* polymorphism in psychiatric disorders.

## Methods and Materials

### Cell Culture

The human neuroblastoma cell line SH-SY5Y (ATCC/CRL-2266) which is near diploid (47 chromosomes) (Spengler et al. 2002) was cultured in a 1:1 mix of Dulbecco’s EMEM and Ham’s F12 media supplemented with 10% (*v*/*v*) fetal bovine serum, 2 mM l-glutamine, 1 mM sodium pyruvate, and penicillin 100 U/ml/streptomycin 0.1 mg/ml. The HAP1 cell lines (Carette et al. 2011; Essletzbichler et al. 2014) were obtained from Horizon Genomics (Cambridge, UK) and cultured in Iscove’s modified Dulbecco’s medium (GIBCO, Paisley, UK), supplemented with 10% (*v*/*v*) fetal bovine serum and 100 U/ml penicillin/0.1 mg/ml streptomycin. Cells were cultured at 37 °C in a humidified 5% CO_2_ atmosphere to 70–80% confluence with culture media being replaced every other day; reagents unless otherwise stated were from Sigma, Dorset, UK.

### Total RNA Preparation and cDNA Synthesis

Total RNA was extracted using Trizol reagent (Invitrogen, Paisley, UK), and 3 μg was reverse-transcribed to cDNA using GoScript® Reverse Transcription System (Promega, Southampton, UK) and random primers following the manufacturers’ instructions.

### Genotyping of the *MAOA* Promoter VNTRs

Genotyping of the *MAOA* uVNTR was performed as previously described (Pickles et al. 2013). The *MAOA* dVNTR PCR reactions (20 μl) contained 10 ng genomic DNA template, 5 pmol of each primer (Forward 5′-FAM-GGGTTAAGCGCCTCAGCTTG-3′ and Reverse 5′-CAAGAGTGGACTTAAGGAAGCAG-3′ [Eurofins, Ebersberg, Germany]), 1× GoTaq® flexi buffer, 1 mM MgCl_2_, 0.1 mM of each dNTP, and 0.625 U of GoTaq® DNA polymerase (Promega, Southampton, UK). 7-DeazaGTP (6.25 μM), 1 M betaine, and 3% (*v*/*v*) DMSO were added to the reaction due to the high GC content of the region. PCR cycling conditions included touchdown to the annealing step from 65 to 55 °C over 10 cycles, followed by 35 cycles at the annealing temperature (55 °C). Analyses were by both 2% agarose gel electrophoresis and by capillary electrophoresis ABI 3130 (Life Technologies, Paisley, UK) in which the genotypes were called using Genemapper V4.0 (Life Technologies) or the QIAxcel Advanced System (Qiagen, Manchester, UK). The results from each method were analyzed blind from each other.

### Horizon Genomics Generation of HAP1 VNTR KO Cells

The HAP1 clones were generated by Horizon Discovery (https://www.horizondiscovery.com/gene-editing/crispr) using the CRISPR/Cas9 deletion system. The KO cell lines were validated by PCR and Sanger sequencing to confirm the presence of the desired mutation at the genomic level (Supplementary Figs. S1–S2).

### mRNA Analysis in the HAP1 Cell Line and KO Derivatives

Reactions containing 10 ng cDNA template, 5 pmol each primer (Eurofins, Ebersberg, Germany), 1× GoTaq® flexi buffer, 1 mM MgCl_2_, 0.1 mM each dNTP, 1 M betaine, and 0.625 U GoTaq® DNA polymerase (Promega, Southampton, UK) were employed to analyze the levels of distinct isoforms of mRNA produced using the following primer pairs: (a) total *MAOA* expression (i.e., isoforms 201 and 204 combined) addressed by amplification of *MAOA* exon III–exon VI fragment Forward 5′-TACGTAGATGTTGGTGGAGCTT-3′, Reverse 5′-AGAATATCCGAGTGGTGCCC-3′; (b) isoform 204 alone *MAOA* exon I–exon IIA fragment, Forward primer 5′-CGGGTATCAAAAGAAGGATCG-3′, Reverse primer 5′-CCAGGAGCTGCTTTCCTCTGATGC-3′. Cycling conditions were 2 min at 95 °C initial denaturation, followed by 35 cycles of 20 s at 95 °C, 20 s at 61 °C and 30 s 72 °C, and final elongation for 5 min at 72 °C.

Amplicons were analyzed using a QIAxcel Advanced System (Qiagen, Manchester, UK,) with the following parameters: QX DNA screening gel cartridge default 2.0 using the AM420 method. Standard alignment marker of 15–600 bp and QX DNA size marker of 25–500 bp were run simultaneously allowing a fully automated size separation and quantification of each sample. Amplicon properties and concentration were determined by QIAxcel BioCalculator software using a proprietary algorithm supplied by the manufacturer. The clonal cell lines were derived from the same parental background and cultured under identical conditions; thus, *MAOA* values were normalized only to the housekeeping gene *β-actin* (Forward primer 5′-CACCTTCTACAATGAGCTGCGTGTG-3′ and Reverse primer 5′-ATAGCACAGCCTGGATAGCAACGTAC-3′) prior to analysis.

### Cohort

Genomic DNA was obtained from saliva of 283 children who participated in the Wirral Child Health and Development Study (WCHADS), a longitudinal Medical Research Council (MRC) funded study of child development. Ethical approval for the study was granted by the Cheshire North and West Research Ethics Committee on the June 27, 2006 (ref: 05/Q1506/107).

#### In Silico Analysis

The University of California Santa Cruz (UCSC) Genome Browser (http://genome.ucsc.edu) was used for genomic positioning and mapping of the selected markers. Genotyped data were subject to quality control examination and gender check. The data was then formatted into a pedigree format file (.PED). MIDAS (Multiallelic Interallelic Disequilibrium Analysis Software) was employed for the LD analysis and construction of the haplotype blocks of the interallelic linkage disequilibrium. The data for both VNTR loci were analyzed following the instructions given by (Gaunt et al. 2006). Common and rare alleles were then imputed based on the inferred haplotype blocks and analysis conducted separately for males and females.

#### Statistical Analysis

The relative gene expression data from the different HAP1 cell lines were analyzed with IBM SPSS Statistics software for Windows, version 24 (IBM Corp., Armonk, NY, USA) through a univariate general lineal model followed by a Bonferroni post hoc test. Data were considered significantly different at *p* < 0.05.

## Results

### Bioinformatic Analysis of *MAOA*

Structured searches through accredited publicly available genomic resources, including UCSC (https://genome.ucsc.edu/), AceView (http://www.ncbi.nlm.nih.gov/ieb/research/acembly/), UniProt (http://www.uniprot.org/), and Ensembl (http://www.ensembl.org/) confirmed four mRNA isoforms for the human *MAOA* gene (201, 202, 203, and 204); two of them (201 and 204) correspond to coding transcripts that predict at least two distinct *MAOA* protein variants (Fig. 1).

The primary *MAOA* isoform (Fig. 1a, isoform 201) comprises a 4015-bp transcript and encodes a full-length protein of 527 amino acids. The 5′UTR of this mRNA isoform was found to extend 124 bp upstream from the first ATG codon with the *MAOA* uVNTR located approximately 1 kb upstream of the TSS used to generate this isoform (Sabol et al. 1998). The second *MAOA* isoform (Fig. 1b; isoform204) had a longer 5′ UTR (~ 1.3 kb), which encompassed the uVNTR within its sequence. This predicted transcript was actually longer than the primary type (5438 versus 4015 bp) and contained an alternative non-coding exon (here termed exon IIA), which would introduce a premature (TGA) stop codon due to a shift in the reading frame. This, in turn, could result in the translational start site shifting to exon IV where the next in-frame methionine codon (ATG) is located, resulting in an amino-terminal truncated version of the MAOA protein (394 AA), exactly 133 residues shorter than the primary isoform as illustrated in Fig. 1b. De Colibus et al. (2005) identified that the *MAOA* FAD/NAD binding domain comprised residues 13–88, 220–294, and 400–462 in the primary isoform therefore an N-terminal section of the FAD/NAD binding domain was omitted in this alternative minor isoform, which incidentally largely overlapped with the non-coding isoform 203 (Fig. 1c).

### Expression of the Two *MAOA* Isoforms in SH-SY5Y Cell Line

**Fig. 2:**
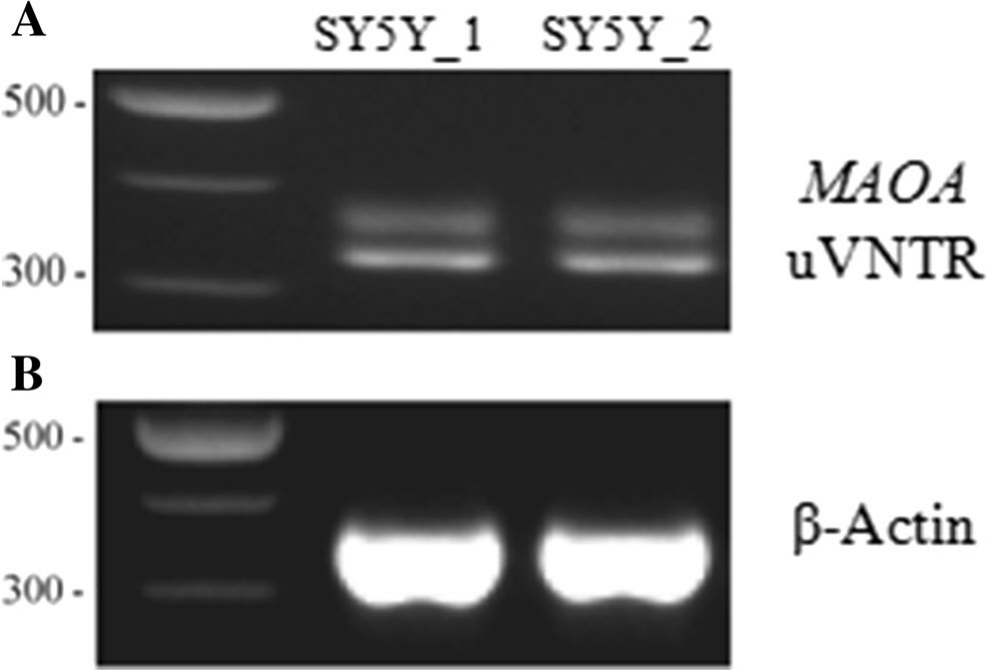
Monoamine oxidase A (*MAOA*) expression in SH-SY5Y cell line. **a** PCR assay using uVNTR primer set demonstrating expression of *MAOA* mRNA under basal conditions. **b** Expression of *β-Actin* in the same SH-SY5Y samples

We used the well-characterized human neuroblastoma female-derived cell line SH-SY5Y which was found to be heterozygous for the uVNTR with three and four copies of the repeat element to address expression of the predicted isoform 204 in extant cells. This allowed identification of allele-specific expression when initiated from the more 5′ TSS that included the uVNTR in the 5′UTR (Fig. 1b, isoform 204). Under basal growth conditions, we detected mRNA corresponding to expression from both alleles using the uVNTR length as the distinguishing feature, thus validating the predicted in silico isoform (Fig. 2a).

### HAP 1 Cell Line Characterization

**Fig. 3:**
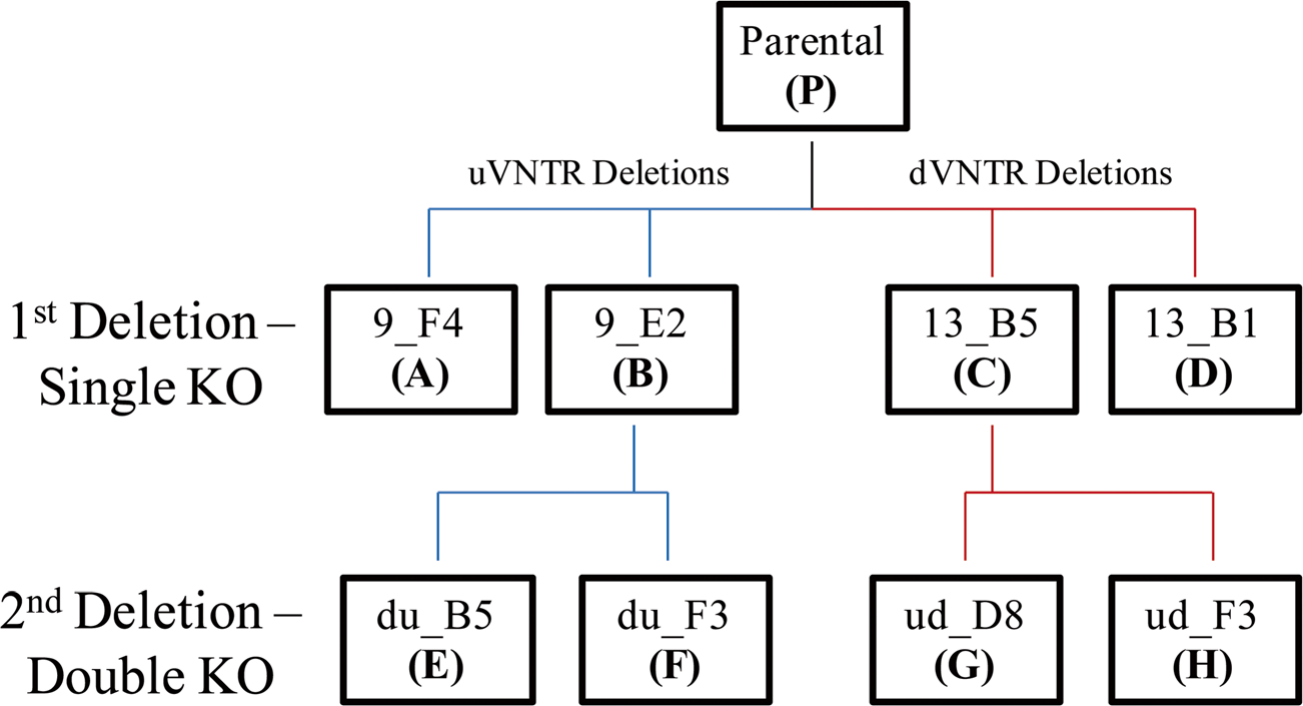
HAP1 cell line genetic tree. Genetic tree illustrating the pedigree of the different HAP1 cell line clones generated. Parental cell line (P) was used to generate the single KO cell lines for uVNTR, on the left side (clones A and B) and for dVNTR on the right side (clones C and D). Clones B and C were used for the generation of a second KO that created the double KO cell lines (E to H) which lack both uVNTR and dVNTR

To address the function of the u- and dVNTRs in *MAOA* gene expression, we utilized CRISPR/Cas9 deletion to produce HAP1cell line clones with selected distinct VNTR haplotypes. As summarized in Fig. 3, four different single KO cell lines were generated from the parental cell line (P) by Horizon Discovery. Two of these had the uVNTR deleted from the *MAOA* promoter region but still possessed the dVNTR positioned upstream of the gene (clones A and B); similarly, two single KO lines were generated targeting the dVNTR initially (clones C and D). To generate the double VNTR KO clones a second, de novo, CRISPR/Cas9 deletion was performed on single KO clones B or C to either remove the dVNTR (clones E and F) or the uVNTR (clones G and H).

The VNTR deletions were confirmed by PCR, individually targeting *MAOA* uVNTR and dVNTR, respectively, as shown in the supplementary Fig. S1. The semi-haploid parental cell line (P) contained the alleles 3R and 10R for the uVNTR and dVNTR, respectively.

### *MAOA* Gene Expression in HAP1 Cells

The expression of the combined putative *MAOA* coding isoforms (i.e., 201 and 204) was assessed using the primer set that amplified the region flanking exon III to exon VI of the *MAOA* gene (Fig. 1) which reported a significant difference of expression between the groups *F*(9,27) = 190.2, *p* < 0.001. Conversely, amplification from exon I to alternative exon IIA allowed us to determine the expression of the less abundant *MAOA* coding isoform 204 alone, which spans the more upstream TSS and encompassed the uVNTR in its 5′UTR (Fig. 1b), which also reported a significant difference of expression among the groups *F*(9,27) = 40.4, *p* < 0.001.

**Fig. 4:**
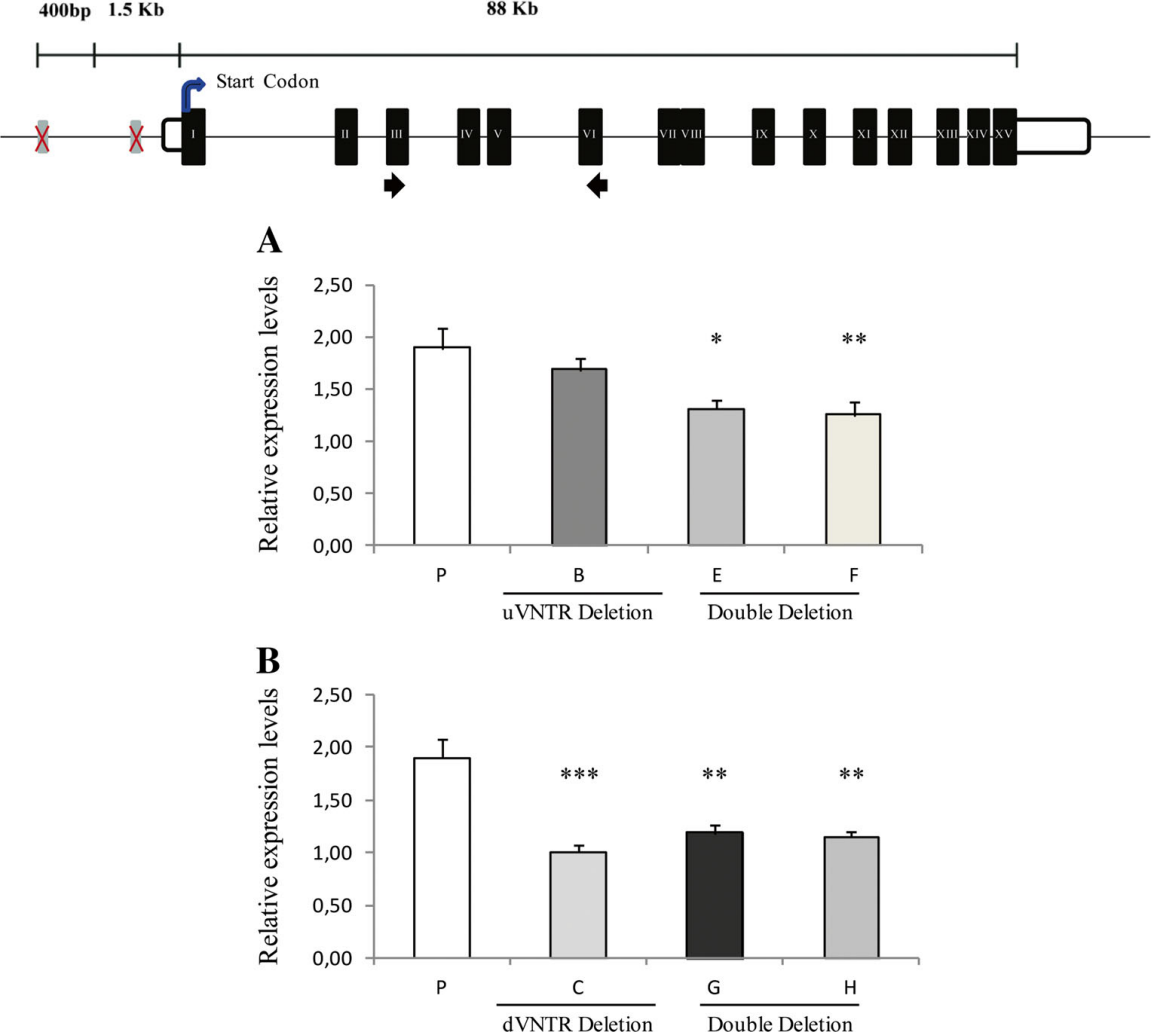
Total monoamine oxidase A (*MAOA*) expression in HAP1 cell lines under basal conditions—single deletion clones and related double KO clones. Illustrated at the top is the *MAOA* gene as reported in UCSC genome browser Hg38 and the most recent version of the Hg19. White boxes represent 5′ and 3′ untranslated regions (UTRs), black boxes the exons, and black straight arrows show the forward and reverse primer sites, respectively. **a** P is the parental cell line, B single uVNTR deletion clone, E (du_B5), and F (du_F3) are the *MAOA* VNTR double KO clones generated from clone. **b** C is the single dVNTR deletion clone, and G and H are the *MAOA* VNTR double KO clones generated from C: ud_D8 and ud_F3, respectively. *p < 0.05, **p < 0.01, ***p < 0.001 of univariate analysis followed by a post hoc Bonferroni test for analyses between more than two groups. All values are expressed as mean ± SEM. For each clone *N* = 4. All values were normalized to *β-actin*

**Fig. 5:**
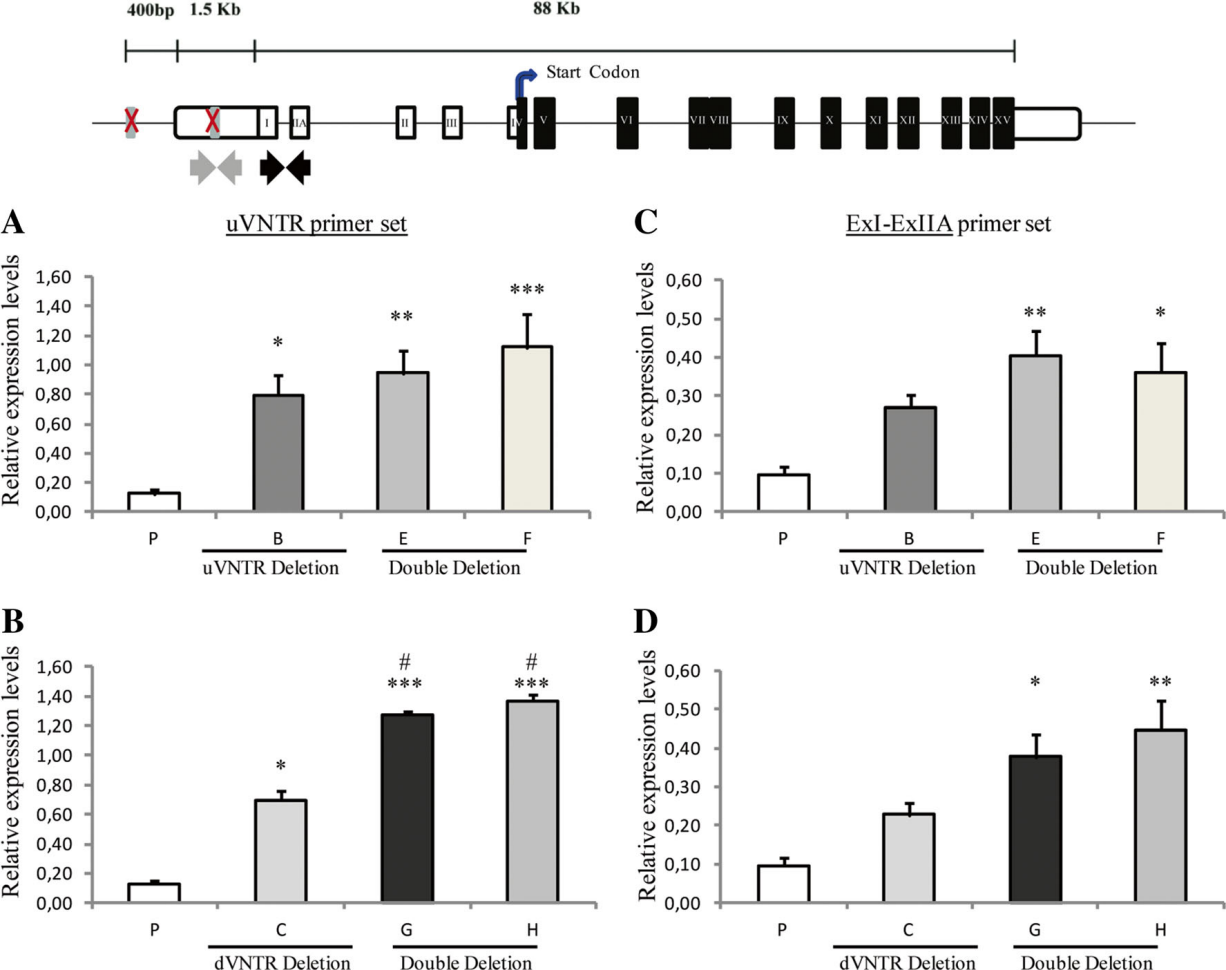
Monoamine oxidase A (*MAOA*) minor isoform expression in HAP1 cell lines under basal condition—single deletion clones and related double KO clones. At the top illustration of *MAOA* gene as reported in UCSC genome browser Hg38 and the most recent version of the Hg19. White boxes represent 5′ and 3′ untranslated regions (UTRs), black boxes the exons. Curved black arrow indicates the translational start site. Black and gray straight arrows show the forward and reverse binding sites for the primers used in analysis. P is the parental cell line, B single uVNTR deletion clone, E(du_B5), and F (du_F3) are the *MAOA* VNTR double KO clones generated from clone B, C single dVNTR deletion clone, and G (ud_D8) and H(ud_F3) are the *MAOA* VNTR double KO clones generated from C. **a**, **b** *MAOA* expression analysis with the uVNTR primer set. **c**, **d** *MAOA* expression analysis with the ExI-ExIIA primer set. ^#^,*p < 0.05, ^##^,**p < 0.01, ^###^,***p < 0.001 of univariate analysis followed by a post hoc Bonferroni test for analyses between more than two groups. Number sign represents significance against the single KO cell line; asterisk represents significance against parental cell line. All values are expressed as mean ± SEM. For each clone *N* = 4. All values were normalized to *β-actin*

The concentration of each isoform was measured using the QIAxcel system after normalization using the housekeeping gene *β-actin*. It was found that the total *MAOA* mRNA (i.e., exon III–exon VI assay) and the alternative isoform 204 alone (i.e., exon I–exon IIA assay) in the parental cell line (P), under basal conditions, had average concentrations of 1.90 and 0.10 ng/μl, respectively (Figs. 4 and 5c, d). This facilitated an estimation of the abundance of the alternative isoform 204 at around 5% of the overall *MAOA* mRNA produced in the parental cell model. Similarly, when using the uVNTR primer set, we obtained the level of isoform 204 at 8% of the total, with a concentration of 0.13 ng/μl (Fig. 5a, b), consistent with the levels obtained with the exon I–IIA assay (Fig. 5c, d).

Deletion of the uVNTR alone from the *MAOA* promoter region did not significantly alter the total *MAOA* expression level when compared to the parental cell line (Fig. 4a, clone B). In contrast, deletion of the dVNTR alone (Fig. 4b, clone C) was sufficient to significantly reduce the expression of total *MAOA* (multiple comparison of Bonferroni post hoc test ***p < 0.01). The expression directed by the double KO cell lines in both Fig. 4a, b (clones E, F, G, and H) was comparable to that of the single dVNTR KO alone. The expression analysis for each single KO clone for both the uVNTR (clones A and B) and dVNTR (clones C and D), as well as the double KO clones (clones E, F, G and H) are contained in supplementary Figs. S3, S4, and S5, respectively.

We next addressed the regulation of expression of the minor isoform 204 by amplification of exons I–IIA (Fig. 5c, d). The parental cell line (P) expressed only a low level of this isoform (~ 5% of total mRNA); however, in comparison, the clones with single deletions of either the u- (Fig. S6-B, clones A and B) or dVNTR (Fig. S7-B, clones C and D) had higher levels of expression ~ 3-fold increase and ~ 2-fold increase, respectively. Furthermore, the data derived from the double KOs derived from clone C (clones G and H, i.e., –d then –u) (Fig. 5d) was supportive of an additive effect on the expression of this *MAOA* isoform. Although the same trend was observed for clones derived from clone B (clones E and F, i.e., –u then –d) (Fig. 5c), the increment was not statistically significant compared to their parental clone B. The expression analysis data for this *MAOA* isoform for the single KO clones for the uVNTR and dVNTR is provided in supplementary Figs. S6 and S7, respectively. These findings were replicated using the primer set targeting the uVNTR (*F*(9,27) = 66.4, *p* < 0.001) in the 5′UTR of this isoform (Fig. 5a, b), where a similar pattern to that obtained with the exon I–exon IIA primer set was observed, namely all comparisons of isoform 204 between the double KO clones (E, F, G, and H) and the parental cell line (P) were found to be significant with an increase in expression (Fig. 5a–d).

### The Haplotype of Distal and Proximal VNTRs in the Population

**Fig. 6:**
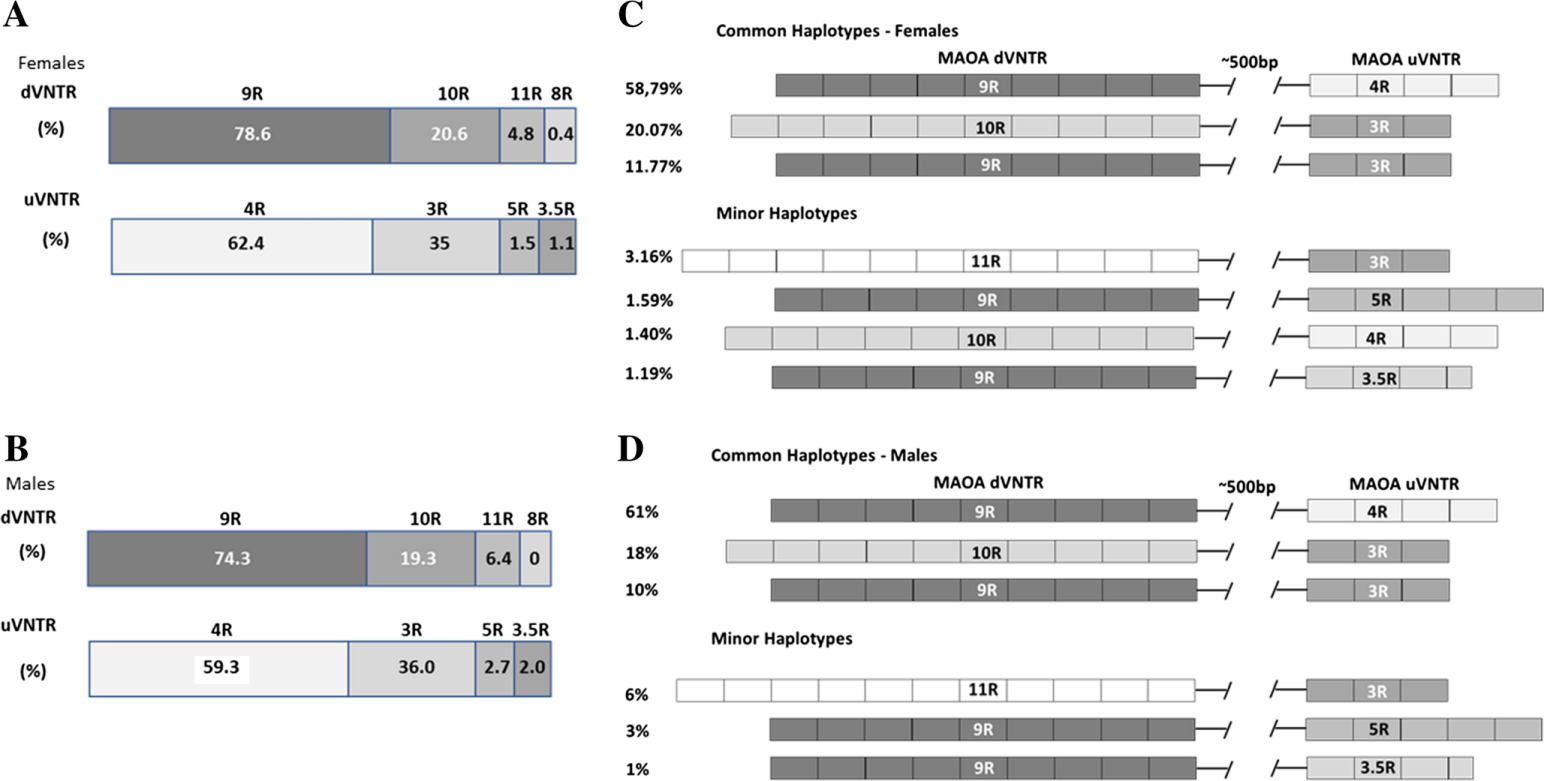
Allele frequencies (**a**, **b**) and haplotype distribution (**c**, **d**) in the Wirral child health and development study (WCHADS, *n* = 283). Assuming a multiallelic system for both markers, the haplotype constructs, frequency, and distribution are illustrated above (*n* = 262). Haplotypes were initially inferred from males and then the Midas package (Gaunt et al. 2006) was used to work out frequencies in females. Hardy-Weinberg equilibrium (HWE) test for females assumed a biallelic system (*p* = 0.82 for uVNTR; and *p* = 0.02 for dVNTR). NB* HWE test was not conducted for males due to the locus hemizygosity

As they have different regulatory properties, analyzing the haplotype block containing the d- and uVNTRs may allow for further stratification and consequently improve genetic associations which otherwise would be solely based on the genotype of the uVNTR. The allele frequencies for each VNTR locus are shown in Fig. 6. Further to this and given the multi-allelic nature of these two loci, we tested both markers for Hardy-Weinberg equilibrium (HWE) within the female arm, accounting only for the common alleles in a bi-allelic system (*p* = 0.82 for dVNTR and *p* = 0.02 for uVNTR) (HWE test was not conducted for males due to the locus hemizygosity). Next, we employed an analysis tool for the construction of the haplotype blocks of the interallelic linkage disequilibrium that accounted for poly-allelic markers (MIDAS, Multiallelic Interallelic Disequilibrium Analysis Software) (Gaunt et al. 2006). Common and rare alleles were then imputed based on the inferred haplotype blocks, and separate analysis for males and females was conducted. This enabled an assessment of the expected versus the observed frequency of both common and rare VNTR haplotypes assuming independent segregation (Table 1). All the haplotype blocks containing the two common alleles for each locus (i.e., 9R and 10R for dVNTR; and 4R and 3R for uVNTR) significantly deviated from their expected haplotype frequencies, and this was highlighted by the significant adjusted *χ*^2^ values observed. This extended previous work by Philibert et al. (2011), demonstrating the existence of significant linkage disequilibrium (LD) between these two loci, suggests a lack of recombination between them, with the alleles likely to segregate as part of the same block. Indeed, only three of the four possible combination haplotypes were commonly observed, with the haplotype containing the 10R dVNTR and 4R uVNTR alleles being very rare. This is consistent with the dVNTR being a more recent polymorphism than the uVNTR and that the minor 10R variant of dVNTR most likely had arose from the same strand containing the minor 3R allele of uVNTR (Supplementary Fig. S8). Therefore, this LD and haplotype analysis allowed us to place the 4R uVNTR with the 9R dVNTR allele and stratify the 3R uVNTR allele with either a 10R, 9R, or, to a lesser extent, the 11R dVNTR. Therefore, in summary, the haplotype comprising both major alleles (9R-4R) was the most common one (59.13% females, 60.29% males), followed by 10R-3R (20.07% females, 18.38% males) and 9R-3R (11.77% females, 9.56% males), with the observed 10R-4R haplotype frequency at around 1% only (against an expected frequency above 10%). The rare 3.5R and 5R uVNTR alleles were both exclusively linked with the common 9R dVNTR, whereas the 11R dVNTR was more often part of the block containing the variant 3R uVNTR. The 8R allele dVNTR was extremely rare in our samples. Our results demonstrated no significant differences in the distribution of haplotypes between males and females (*t* test *p* > 0.05).

**Table 1:** Distribution test between observed and expected haplotype frequencies

A							B						
dVNTR/uVNTR	Observed frequency (%)	Expected frequency (%)	Yates *χ*^2^	*D*′	*r* ^2^	Haplotype counts	dVNTR/uVNTR	Observed frequency (%)	Expected frequency (%)	Yates *χ*^2^	*D*′	*r* ^2^	Haplotype counts
9R:4R	59.13	45.15	47.8	0.833	0.402	149	9R:4R	60.29	46.05	62.1	0.952	0.481	82
10R:3R	20.07	7.65	47.1	0.901	0.399	51	10R:3R	18.38	6.47	52.4	0.942	0.410	25
9R:3R	11.77	26.22	55.5	− 0.846	0.466	29	9R:3R	9.56	25.37	77.3	− 0.956	0.595	13
11R:3R	3.16	1.70	1.36	0.476	0.024	9	11R:3R	5.88	1.99	13.6	1	0.122	8
9R:5R	1.59	1.17	0.002	1	0.058	4	9R:5R	2.94	2.21	0.34	1	0.01	4
10R:4R	1.19	13.18	41.0	− 0.894	0.348	3	9R:3.5R	2.21	1.38	0.03	1	0.006	3
11R:4R	1.19	2.93	0.87	− 0.492	0.015	3	10R:4R	0.74	11.74	42.0	− 0.937	0.33	1
9R:3.5R	1.19	0.87	0.04	1	0.044	3	10R:3.5R	0.00	0.35	0.001	− 1	0.004	0
8R:3R	0.40	0.14	0.28	1	0.007	1	11R:3.5R	0.00	0.11	n/a	− 1	0.001	0
10R:3.5R	0.00	0.26	n/a	− 0.627	0.001	0	11R:4R	0.00	3.61	10.9	− 1	0.099	0
8R:4R	0.00	0.24	0.31	− 1	0.006	0	10R:5R	0.00	0.56	0.12	− 1	0.007	0
8R:5R	0.00	0.01	n/a	− 1	6.00E-05	0	11R:5R	0.00	0.17	n/a	− 1	0.002	0
10R:5R	0.00	0.34	0.02	− 1	0.044	0							
11R:5R	0.00	0.08	n/a	− 1	0.008	0							
8R:3.5R	0.00	0.00	n/a	− 1	5.00E-05	0							
11R:5R	0.00	0.06	n/a	− 1	0.0006	0							

(A) Females (2*n* = 252) and (B) males (*n* = 126). Assuming a multiallelic system, adjusted *χ*^2^ values were derived from the observed versus expected frequencies based on the allele frequencies obtained for this study. Frequencies and respective *D*′ and correlation *r*^2^ values for each haplotype depicted above were calculated using the MIDAS package (Gaunt et al. 2006)

*n/a* there are no observed frequencies for that haplotype in our cohort

## Discussion

Transcriptomic mapping using Hg38 and ENCODE data indicated that there are multiple isoforms for the *MAOA* gene (Fig. 1), which was confirmed by mRNA expression data in this study. These two isoforms could be easily distinguished at several levels. The “canonical” *MAOA* isoform, which we termed the primary isoform (Fig. 1, isoform 201), comprised 15 exons with a shorter 5′ UTR. Conversely, the alternative isoform (Fig. 1, isoform 204) had 16 exons and a longer 5′ UTR, which contained the commonly reported uVNTR (Sabol et al. 1998). Our in silico analysis suggested that the extra exon present in this latter isoform, here termed exon IIA, would introduce a TGA stop codon, thus potentially causing a shift in the start codon for protein translation to exon IV (Fig. 1b). Our study investigated the role of two VNTRs in the *MAOA* promoter in directing expression from each of the TSS. To address this, we exploited the near-haploid cell line HAP1, which was engineered to remove either the uVNTR, the dVNTR, or both from the *MAOA* promoter sequence. This allowed us to assess, separately or in combination, the expression patterns of the distinct *MAOA* isoforms in the presence or absence of these two elements.

Under basal growth conditions in vitro, deletion of the uVNTR did not significantly modulate expression of total *MAOA* mRNA (Fig. 4a). Conversely, the dVNTR deletion significantly reduced expression of total *MAOA* mRNA (Fig. 4b), demonstrating that it was a positive regulator of the primary *MAOA* isoform, which has not been previously reported. Next, we assessed the impact of the double VNTR KOs and found our results to be in line with the mode of action of the single KOs. Specifically, the expression of the primary *MAOA* isoform in the double KO cell lines appeared to be significantly lower than the parental cell line (Fig. 4) with the major mediator being the dVNTR. Indeed, when the expression of the double KO clones was compared to the single dVNTR deletion, we did not find any significant differences (Fig. 4b). Our data therefore support a major role for the dVNTR in driving the expression of the primary *MAOA* isoform, further illustrated by the significant reduction of this isoform seen in the double KOs on the uVNTR KO background (Fig. 4a). In contrast, we demonstrated that deletion of either the d- or u- VNTR resulted in increased expression of the minor alternative isoform 204, which contained the uVNTR within its 5′ UTR (Fig. 5a–d); therefore, both VNTRs individually appeared to act as negative regulators of this isoform. The double VNTR KOs also substantially increased the expression of this transcript in relation to the parental cell line (P) (Fig. 5c, d). These observations were replicated using an independent PCR assay targeting the uVNTR (Fig. 5a, b).

Our data indicated that the haplotype of the d- and uVNTRs could account for significant differential regulation of *MAOA* expression. This would be consistent with previous data showing that distinct combinations of the d- and uVNTRs (9R:3R and 9R:4R) supported distinct reporter gene expression patterns (Philibert et al. 2011). The DNA sequences of the d-and u-VNTR are quite distinct and are therefore likely to bind a different set of transcription factors and mediate a differential response to the same challenge as we and others have demonstrated for the well-characterized regulatory VNTR elements in the *SLC6A4* and *SLC6A3* genes (Michelhaugh et al. 2001; Vasiliou et al. 2012). Our data indicated that the uVNTR and dVNTR are parts of the same haplotype block; consequently, several CNS disorders and conditions solely attributed to the uVNTR could in fact be mechanistically associated with the dVNTR. However, confirmation of this hypothesis will require profiling of well-characterized cohorts larger than the WCHADS cohort we used to address the frequency of these VNTRs in the population.

## Conclusions

**Fig. 7:**
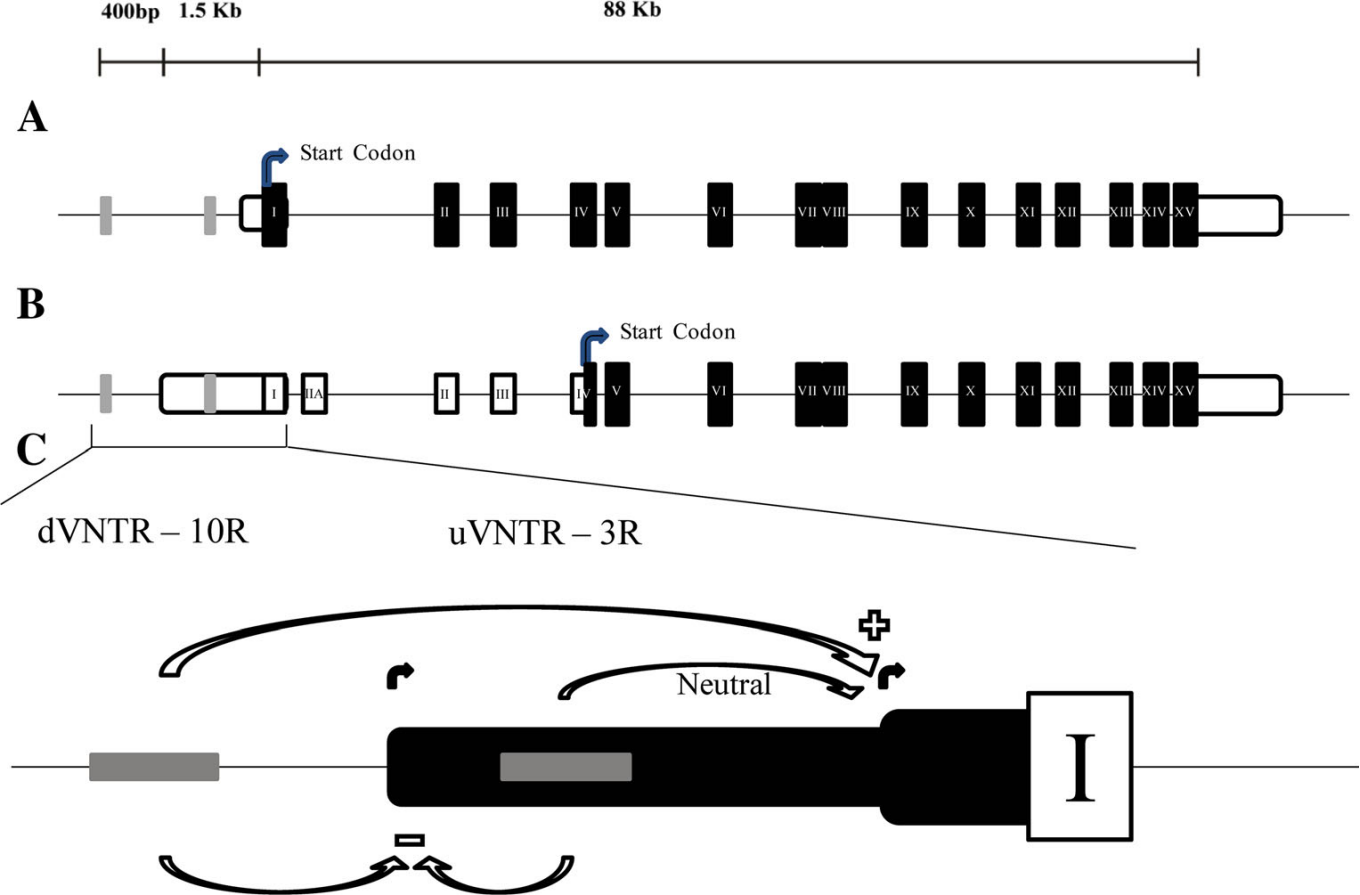
Regulation model for monoamine oxidase A (*MAOA*) expression by the u- and dVNTRs. Graphic representation of the *MAOA* promoter region and the regulatory effect exerted by the u- and dVNTRs on the two *MAOA* isoforms. White box represent exon I, gray bars represent *MAOA* dVNTR and uVNTR from left to right respectively, and overlapped black boxes represent 5′UTRs of the two isoforms. Curved black lines represent the transcriptional start sites (TSSs) for the two *MAOA* isoforms

Taken altogether, these results give further insights into the complexity of *MAOA* regulation, which historically has focused on the uVNTR element to account for variations in the expression of this gene and risk to neuropsychiatric conditions. Here we provide evidence that this is only a partial explanation, the dVNTR plays a significant role in MAOA expression, and our work supports mechanistic interactions between these two elements. Indeed, we have confirmed that deletion of both elements did not preclude expression of the gene itself, although the level of mRNA transcripts was significantly reduced compared to the parental cell line. The dVNTR appeared to be a major positive regulator of the primary transcript isoform, whereas both VNTRs seemed to act in concert to negatively modulate the expression of the minor transcript isoform 204 (Fig. 7). Our data pertains to the HAP1 cell line which was chosen as it expressed both isoforms of MAOA and its haploid karyotype facilitated successful CRISPR deletion of regulatory elements. Expression of MAOA will vary in a tissue specific and stimulus inducible manner and regulation in a neuronal context may differ; however, our model supplies a framework to incorporate both VNTRs in MAOA regulation. Both VNTRs are primate specific, which suggests that there are additional key regulatory domains for *MAOA* expression in mammals as a whole. Furthermore, the uVNTR could have a dual function in both transcription and post-transcriptional regulations of the *MAOA* gene as it is contained within the 5′UTR of one of the putative coding isoforms. Such a dual function has been demonstrated for a VNTR in the mir137 gene depending on the mRNA isoform that was expressed (Warburton et al. 2016). In conclusion, we believe that our data provides significant insights into the understanding of the regulation of *MAOA* expression and its modulation by genetic variants.

## Electronic Supplementary Material

ESM 1 (DOCX 14863 kb)
