# Supplementary material for: The Regulation of Monoamine Oxidase A Gene Expression by Distinct Variable Number Tandem Repeats
Source: J Mol Neurosci. 2018 Mar 14;64(3):459–70. doi: 10.1007/s12031-018-1044-z (PMC5874270; doi:10.1007/s12031-018-1044-z)
Supplement: Supplementary file 1 — (DOCX 14863 kb) [file 12031_2018_1044_MOESM1_ESM.docx]

**Supplementary material**

**Journal of Molecular Neuroscience**

**The regulation of Monoamine Oxidase A gene expression by distinct Variable Number Tandem Repeats**

Maurizio Manca ^1,2§#^, Veridiana Pessoa ^1,2§+^, Ana Illera Lopez ^1^, Patrick T Harrison ^6^, Fabio Miyajima ^1+^, Helen Sharp ^2^, Andrew Pickles ^3^, Jonathan Hill ^4^, Chris Murgatroyd ^5^, Vivien J Bubb ^1^, John P Quinn ^1*^

1. Department of Molecular and Clinical Pharmacology, Institute of Translational Medicine, University of Liverpool, Liverpool L69 3BX, UK
2. Institute of Psychology, Health and Society, University of Liverpool, Liverpool, UK
3. King’s College London, MRC Social Genetic and Developmental Psychiatry Research Centre, Institute of Psychiatry, London, UK
4. School for Psychology and Clinical Language Sciences, University of Reading, Reading, UK
5. School of Healthcare Science, Manchester Metropolitan University, Manchester, UK
6. Department of Physiology, BioSciences Institute, University College Cork, Cork, Ireland

§ These authors contributed equally to this work

+ Current address; Drug Development and Research Center, Department of Physiology and Pharmacology, Faculty of Medicine - Federal University of Ceara, Brazil

# Current address: Manchester Pharmacy School, University of Manchester, Stopford Building, Oxford Road, Manchester, M13 9PT, UK

*Corresponding author: John P. Quinn, Department of Molecular and Clinical Pharmacology, The University of Liverpool, Liverpool, UK, L69 3BX. Tel: +44 151 794 5498.

E-mail: [jquinn@liverpool.ac.uk](mailto:jquinn@liverpool.ac.uk)


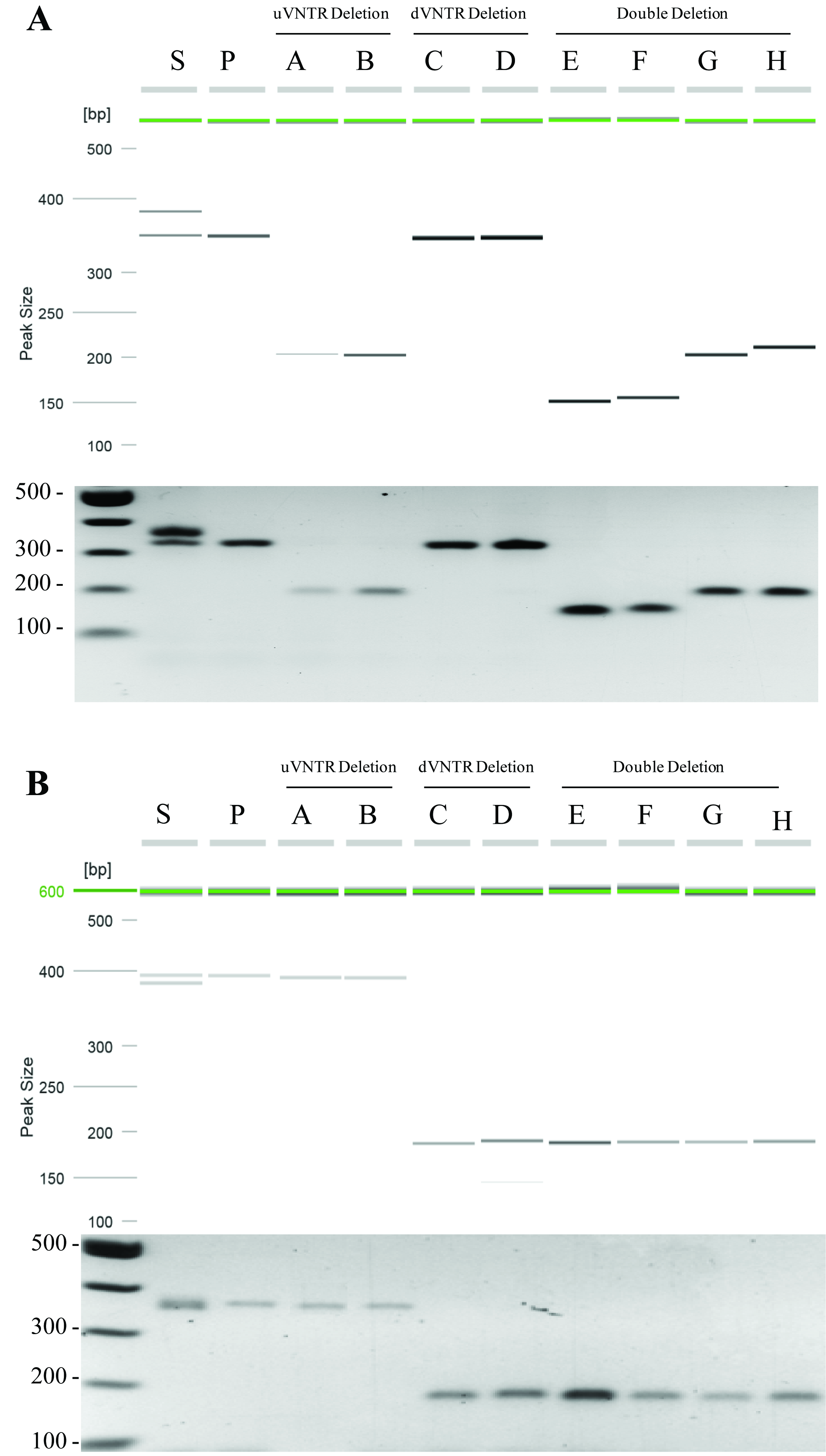


Figure S1 - HAP1 cell line genotype**.** Genotype of HAP1 cell line clones (A-H) confirming the deletion of the genetic region of interest. **A** - PCR reaction amplifying uVNTR genetic region. **B** - Reaction amplifying dVNTR genetic region. Letters on top refer to Figure 3 for the clones, S is SH-SY5Y neuroblastoma cell line P corresponds to parental cell line.

This analysis confirmed that the DNA deletions had been successful and the actual PCR product sizes corresponded to the expected ones (data file information from Horizon). However, a substantial difference of 43 bp was observed between clones E, F and clones G, H at the uVNTR locus whilst there was no difference in the dVNTR one.


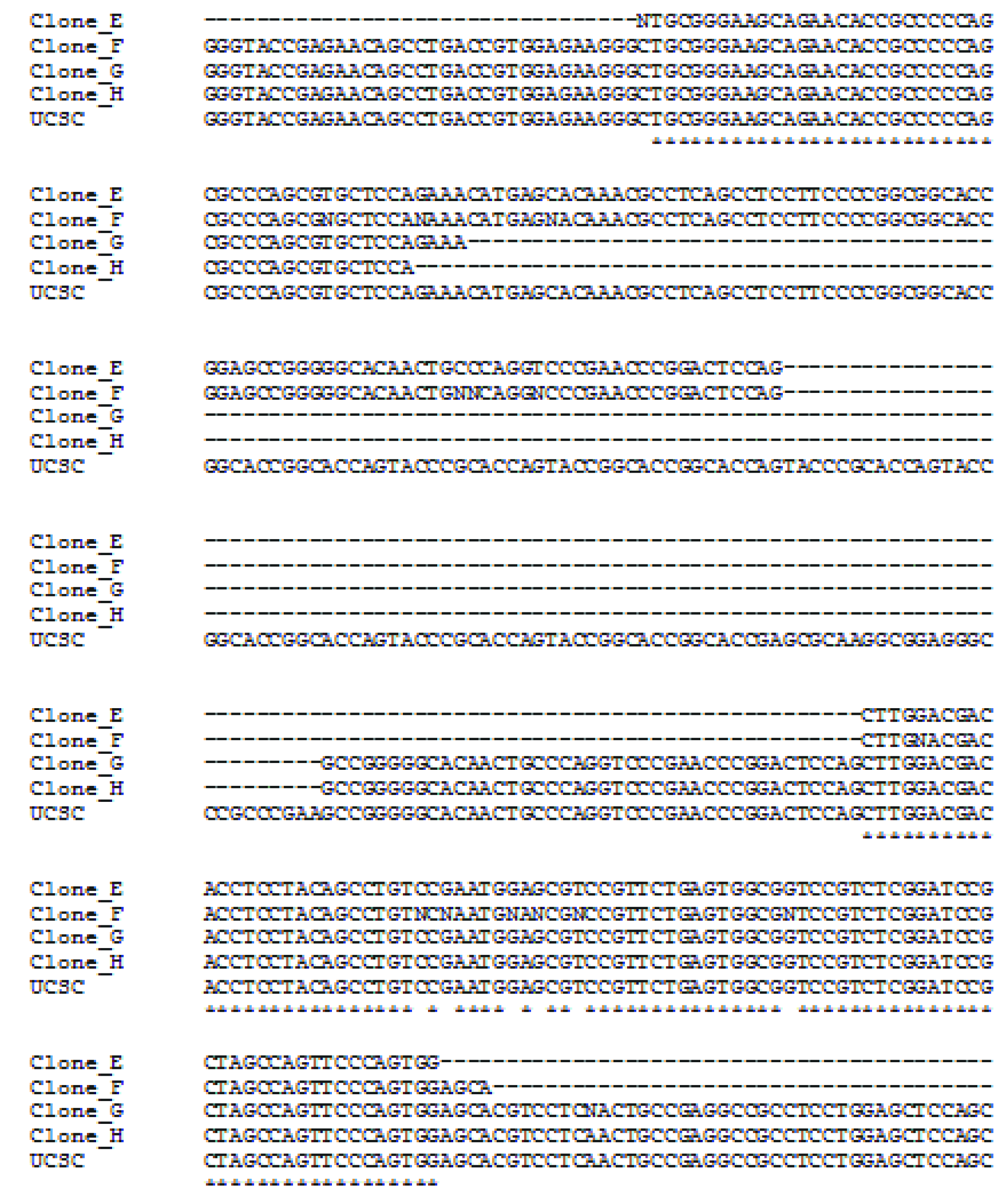


Figure S2 - HAP1 double KO alignment**.** Alignment of the HAP1 double KO clones E, F, G and H after sequencing of each clone.


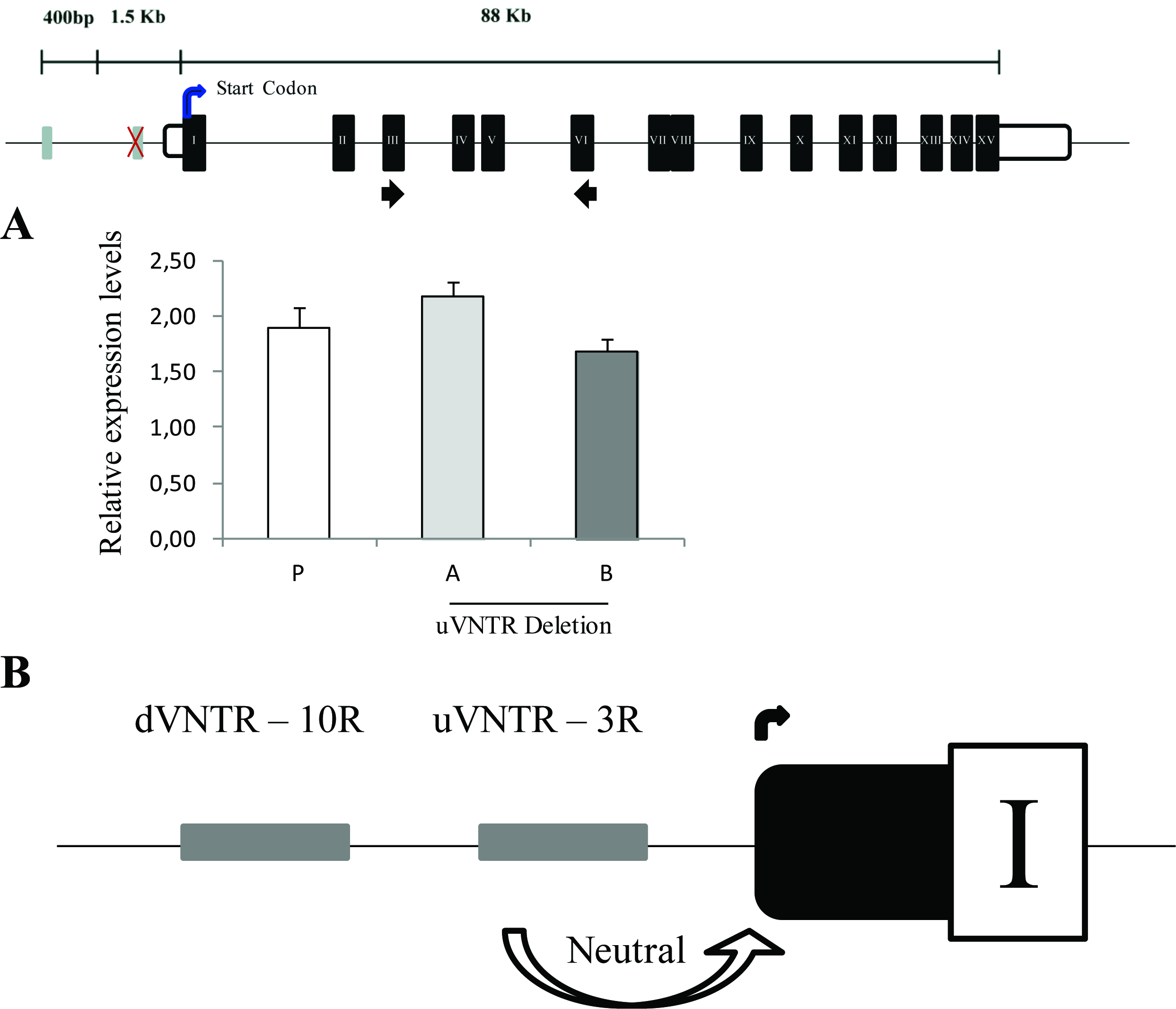


Figure S3 - Monoamine oxidase A (*MAOA*) expression in HAP1 cell line – uVNTR deletion clones**. A.** Relative expression level of primary *MAOA* mRNA (201) under basal conditions. At the top, illustration of *MAOA* gene as reported in UCSC genome browser Hg38 and the most recent version of the Hg19 (GENECODE v24 track). White boxes represent 5ʹ and 3ʹ untranslated regions (UTRs), black boxes the exons. Curved black arrow indicates the transcription start site (TSS). Black straight arrows show the forward and reverse primers respectively. P is the parental cell line, A and B are uVNTR single deletion clones: 9_F4 and 9_E2 respectively. All values are expressed as mean ± SEM. For each clone N=4. All values were normalized to β-Actin. **B.** Illustration of the *MAOA* gene and the effect on the primary isoform expression of the uVNTR.


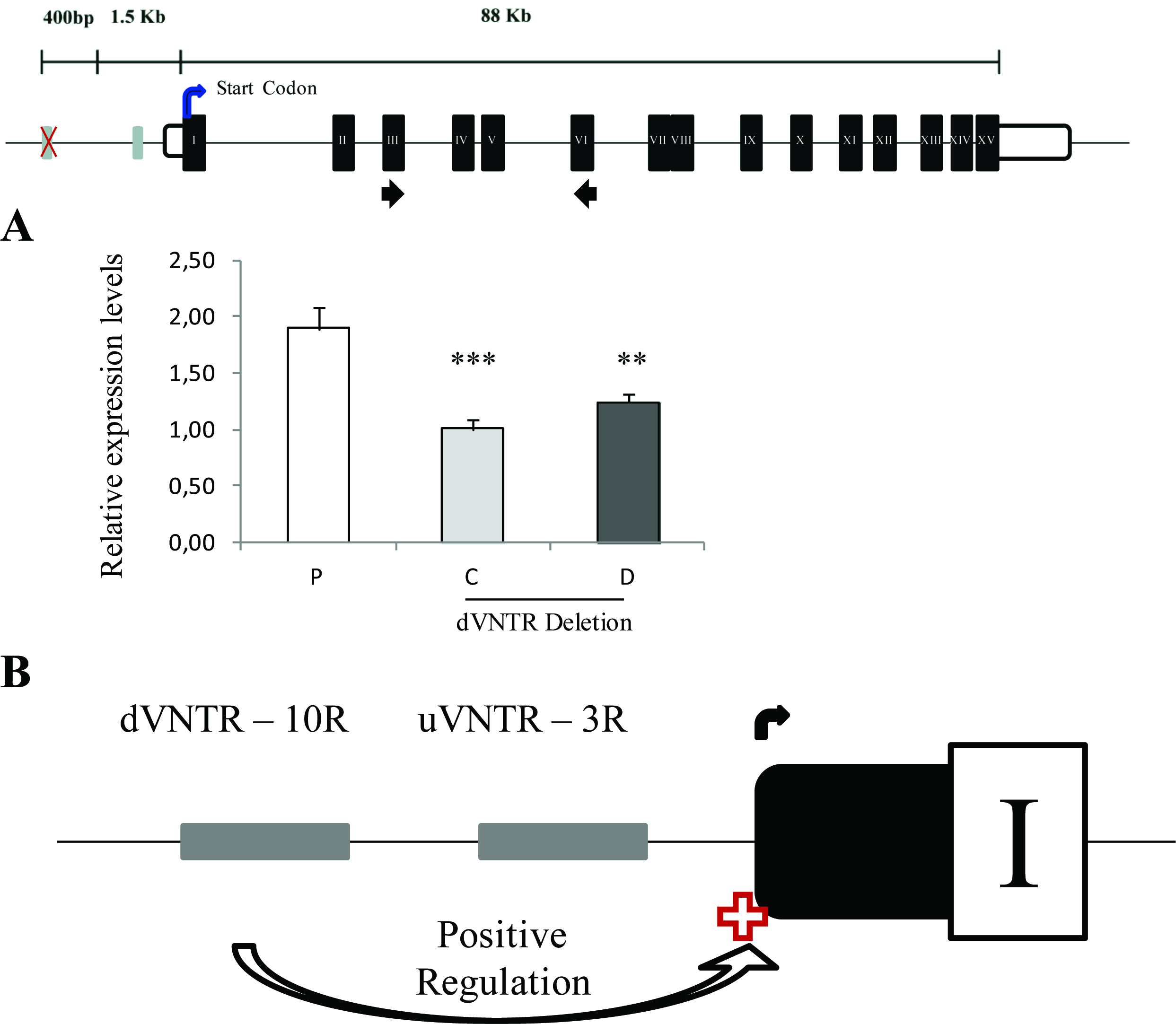


Figure S4 - Monoamine oxidase A (*MAOA*) expression in HAP1 cell line – dVNTR deletion clones**. A.** Relative expression level of primary *MAOA* (201) mRNA under basal conditions. At the top, illustration of *MAOA* gene as reported in UCSC genome browser Hg38 and the most recent version of the Hg19 (GENECODE v24 track). White boxes represent 5ʹ and 3ʹ untranslated regions (UTRs), black boxes the exons. Curved black arrow indicates the transcription start site (TSS) for the full-length MAOA protein. Black straight arrows show the forward and reverse primers respectively. P is the parental cell line, C and D are the dVNTR single deletion clones: 13_B5 and 13_B1 respectively. *p<0.05, **p<0.01, ***p<0.001 of univariate analysis followed by a post hoc Bonferroni test for analyses between more than two groups. All values are expressed as mean ± SEM. For each clone N=4. All values were normalized to β-Actin. **B.** Illustration of the *MAOA* gene and the effect on the primary mRNA isoform (201) expression of the dVNTR. Curved black line represents the TSS.


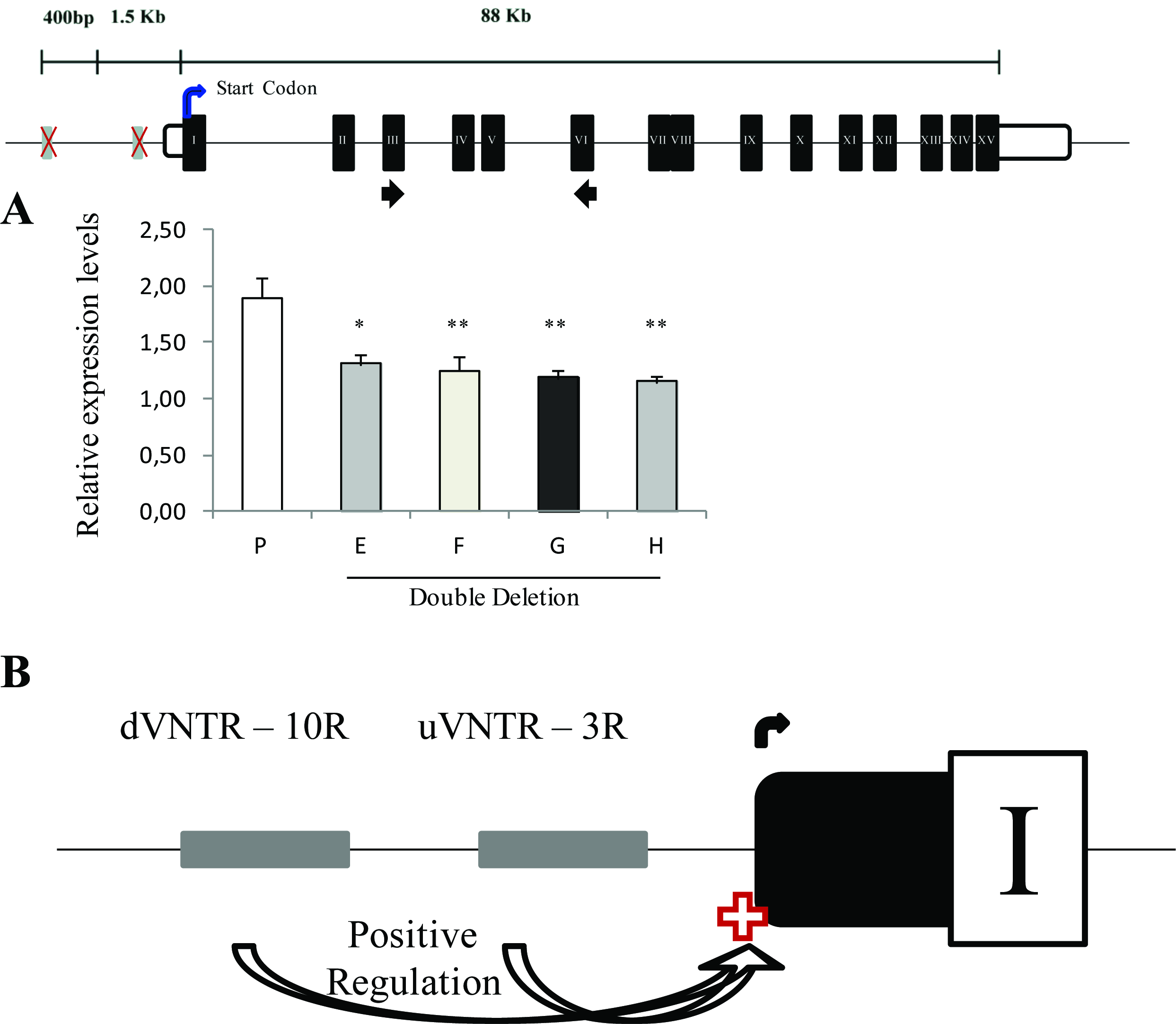


Figure S5 - Monoamine Oxidase A (*MAOA*) expression in HAP1 cell line – double deletion clones**. A.** Relative expression level of primary *MAOA* mRNA under basal conditions. At the top, illustration of *MAOA* gene as reported in UCSC genome browser Hg38 and the most recent version of the Hg19 (GENECODE v24 track). White boxes represent 5ʹ and 3ʹ untranslated regions (UTRs), black boxes the exons. Curved black arrow indicates the transcription start site (TSS) for the full-length MAOA protein. Black straight arrows show the forward and reverse primers respectively. P is the parental cell line, E, F, G and H are the MAOA VNTRs double KO clones: du_B5, du_F3, ud_D8 and ud_F3 respectively. *p<0.05, **p<0.01, ***p<0.001 of univariate analysis followed by a post hoc Bonferroni test for analyses between more than two groups. All values are expressed as mean ± SEM. For each clone N=4. All values were normalized to β-Actin. **B.** Illustration of the *MAOA* gene and the effect on the primary mRNA isoform expression of the *MAOA* VNTRs. Curved black line represents the TSS.


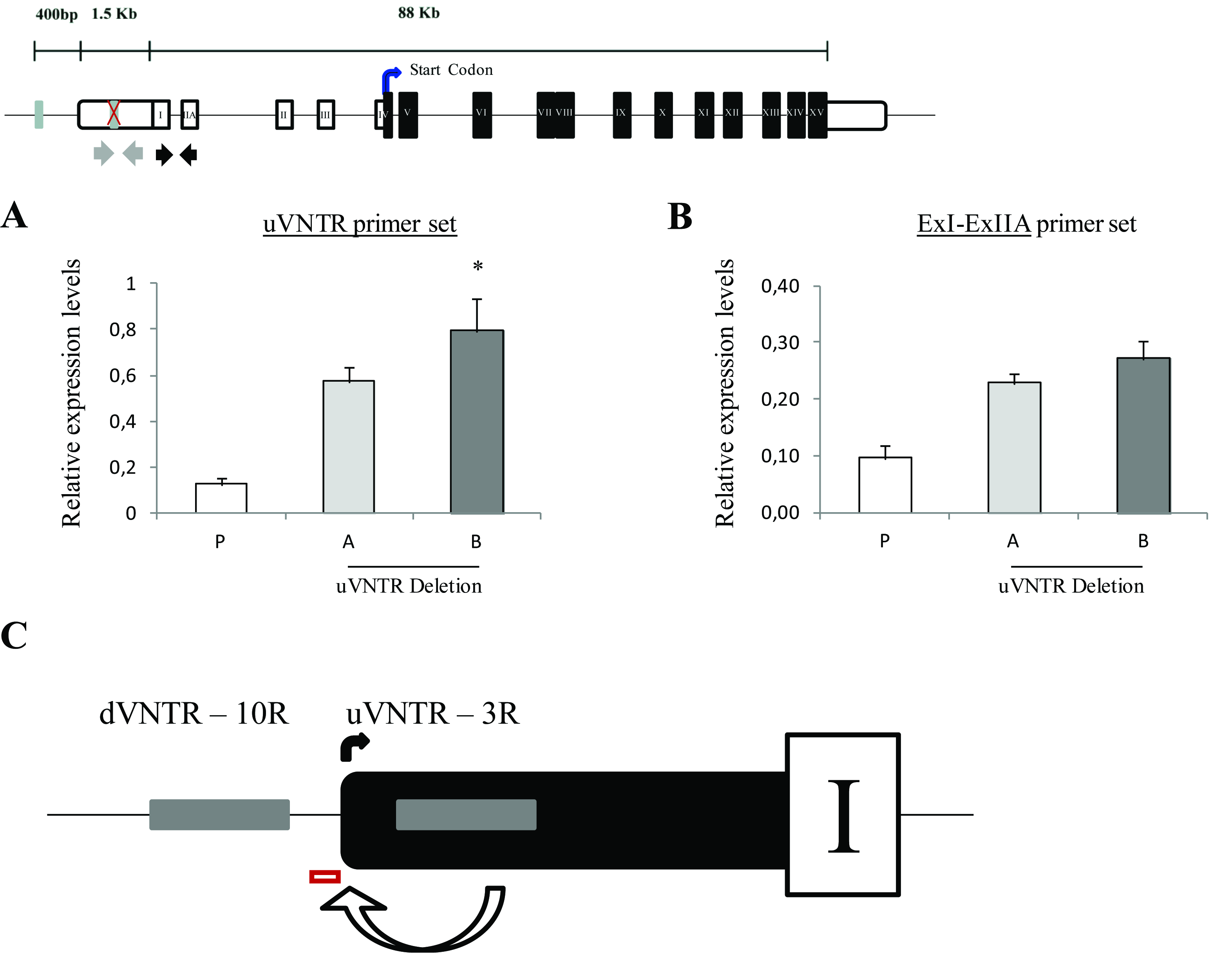


Figure S6 - Monoamine oxidase A (*MAOA*) secondary isoform expression in HAP1 cell line – uVNTR deletion clones**.** Relative expression levels of secondary mRNA (204) under basal conditions, At the top, illustration of *MAOA* gene as reported in UCSC genome browser Hg38 and the most recent version of the Hg19. White boxes represent 5ʹ and 3ʹ untranslated regions (UTRs), black boxes the exons. Curved black arrow indicates the transcription start site (TSS) for the secondary MAOA mRNA (204). Black and grey straight arrows show the forward and reverse primer sets used. P is the parental cell line, A and B are the uVNTR single deletion clones: 9_F4 and 9_E2 respectively. **A**. Analysis with the uVNTR primer set. **B**. Analysis with the ExI-ExIIA primer set. *p<0.05, **p<0.01, ***p<0.001 of univariate analysis followed by a post hoc Bonferroni test for analyses between more than two groups. All values are expressed as mean ± SEM. For each clone N=4. All values were normalized to β-Actin. **C.** Illustration of the *MAOA* gene and the effect of the uVNTR on the minor isoform (204) expression. Curved black line represents the TSS.


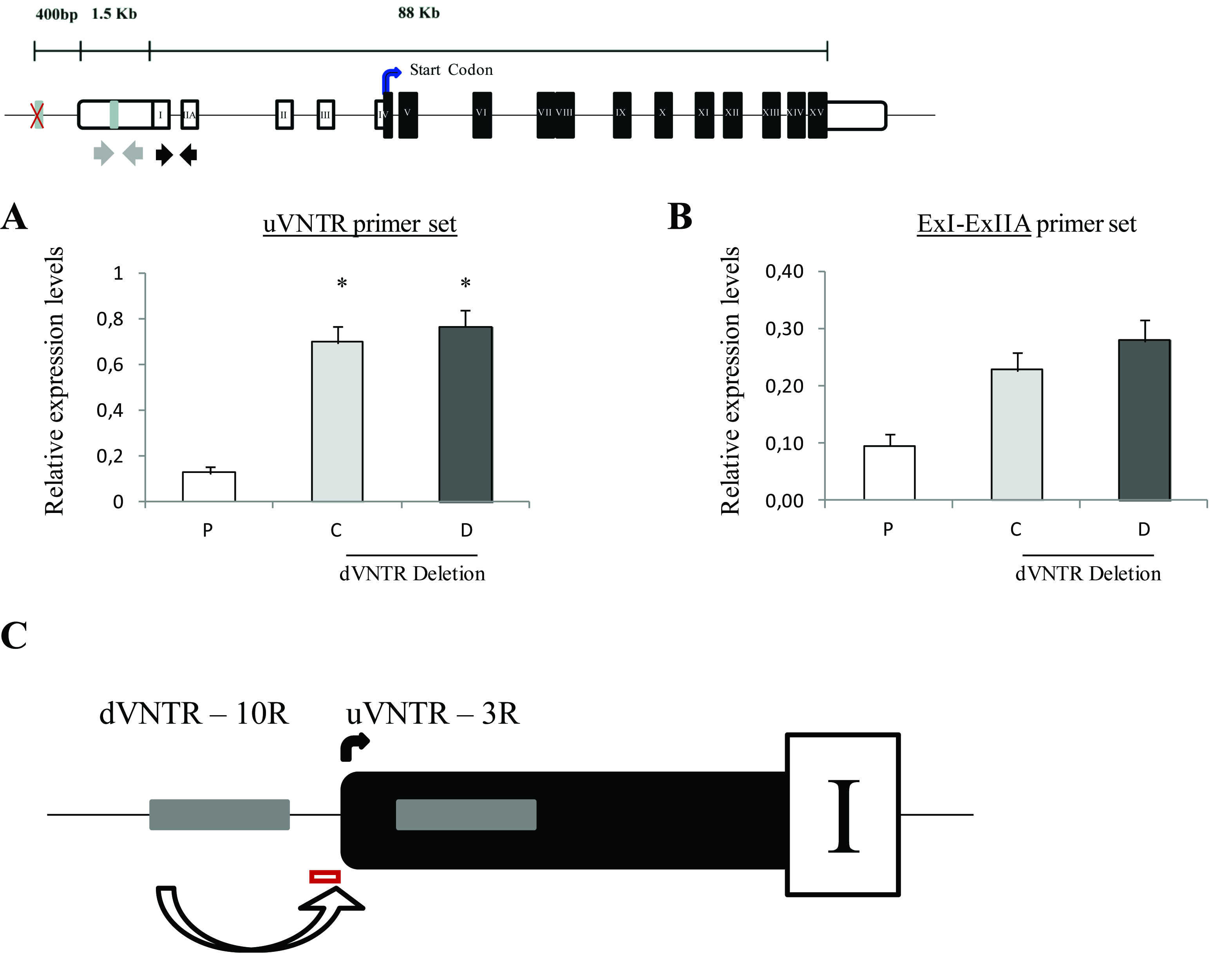


Figure S7 - Monoamine oxidase A (*MAOA*) minor isoform expression in HAP1 cell line – dVNTR deletion clones**.** Relative expression level of secondary *MAOA* mRNA (204) under basal conditions. At the top, illustration of *MAOA* gene as reported in UCSC genome browser Hg38 and the most recent version of the Hg19. White boxes represent 5ʹ and 3ʹ untranslated regions (UTRs), black boxes the exons. Curved black arrow sets the transcription start site (TSS) for the minor/secondary MAOA mRNA. Black and grey straight arrows show the forward and reverse primer sets used.. P is the parental cell line, C and D are the dVNTR single deletion clones: 13_B5 and 13_B1 respectively. **A**. Analysis with the uVNTR primer set. **B**. Analysis with the ExI-ExIIA primer set. *p<0.05, **p<0.01, ***p<0.001 of univariate analysis followed by a post hoc Bonferroni test for analyses between more than two groups. All values are expressed as mean ± SEM. For each clone N=4. All values were normalized to β-Actin. . **C.** Illustration of the *MAOA* gene and the effect on the minor isoform (204) expression of the uVNTR. Curved black line represents the TSS.

Similarly as observed for the uVNTR KO clones (Figure S6A), the expression of both dVNTR KO clones, C and D, was significantly higher than the parental cell line

The deletion of either the uVNTR or the dVNTR seem sufficient to increase the expression of the MAOA secondary mRNA isoform (204) highlighting the possibility of a negative regulatory element in both VNTRs specific for this mRNA isoform, this was in contrast with the primary mRNA isoform (201) where only the dVNTR appeared to modulate expression.

**
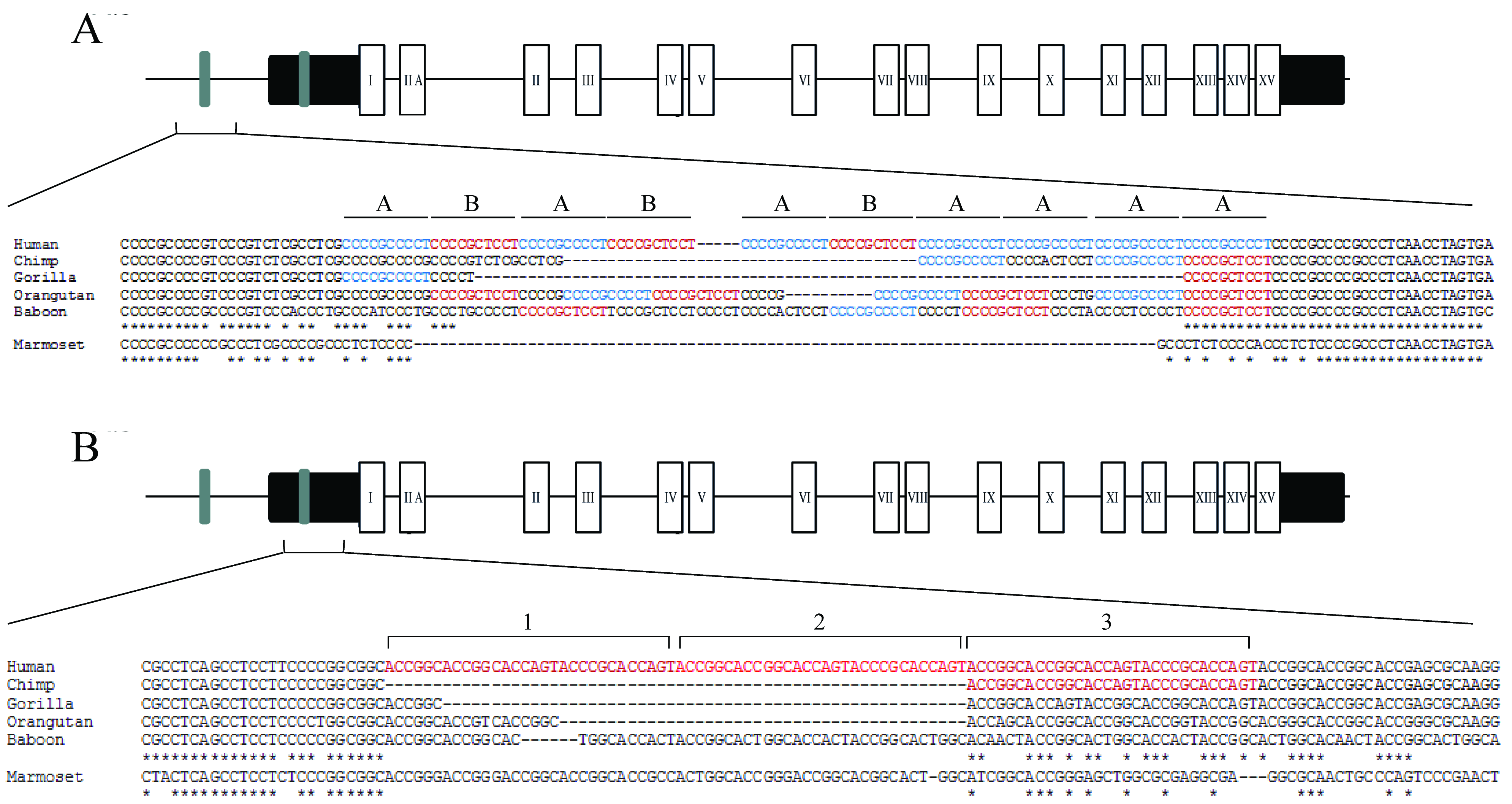
**

Figure S8 – Monoamine oxidase A (*MAOA)* VNTRs conservation in human and non-human primates. **A.** Conservation analysis of the *MAOA* dVNTR. At the top graphic illustration of the *MAOA* gene. Underneath is the alignment of the sequences for humans and non-human primates. In blue is highlighted the A decamer of the dVNTR, in red the B decamer. Asterisks show the conserved nucleotides among species. **B.** Conservation analysis of the *MAOA* uVNTR. At the top graphic illustration of the MAOA gene. Underneath the alignment of the sequences for humans and non-human primates. Asterisks show the conserved nucleotides among species.
